# Development of Quinoa Value Chain to Improve Food and Nutritional Security in Rural Communities in Rehamna, Morocco: Lessons Learned and Perspectives

**DOI:** 10.3390/plants10020301

**Published:** 2021-02-05

**Authors:** Abdelaziz Hirich, Sifeddine Rafik, Mohamed Rahmani, Amira Fetouab, Fatima Azaykou, Kaoutar Filali, Hayatullah Ahmadzai, Younes Jnaoui, Aziz Soulaimani, Mariam Moussafir, Mohamed El Gharous, Salwa Karboune, Abdelaziz Sbai, Redouane Choukr-Allah

**Affiliations:** 1 African Sustainable Agriculture Research Institute (ASARI), Mohammed VI Polytechnic University (UM6P), Laayoune 70000, Morocco; 2 Agricultural Innovation and Technology Transfer Center (AITTC), Mohammed VI Polytechnic University (UM6P), Ben Guerir 43150, Morocco; sifeddine.rafik@um6p.ma (S.R.); amirafetouab@gmail.com (A.F.); azaykoufatima@gmail.com (F.A.); younes.jnaoui@um6p.ma (Y.J.); aziz.soulaimani@um6p.ma (A.S.); mariam.moussafir@um6p.ma (M.M.); mohamed.elgharous@um6p.ma (M.E.G.); 3 Department of Food and Nutritional Sciences, Section of Agricultural and Food Industries, Agronomic and Veterinary Medicine Hassan II Institute, Rabat 10112, Morocco; rahmanimohammed@yahoo.fr; 4 Department of Agronomy, Agronomic and Veterinary Medicine Hassan II Institute, Rabat 10112, Morocco; k.filali87@gmail.com (K.F.); asbai2007@gmail.com (A.S.); redouane53@yahoo.fr (R.C.-A.); 5 International Center for Biosaline Agriculture, Directorate of Programs, Dubai 14660, United Arab Emirates; h.ahmadzai@biosaline.org.ae; 6 Department of Food Science and Agricultural Chemistry, Macdonald Campus, McGill University, Sainte-Anne-de-Bellevue, Québec, QC H9X 3V9, Canada; salwa.karboune@mcgill.ca

**Keywords:** production cost, pearling, yield, irrigation, mechanization, harvest

## Abstract

Agricultural production in the Rehamna region, Morocco is limited with various challenges including drought and salinity. Introduction of climate resilient and rustic crops such as quinoa was an optimal solution to increase farmer’s income and improve food security. This study summarizes results obtained from a research project aiming to develop quinoa value chain in Morocco. The study tackled several aspects including agronomic traits (yield and growth), transformation, quality (nutritional and antinutritional traits) and economic analysis and, finally, a strength–weaknesses–opportunities–threats analysis, lessons learned and development perspectives were presented. From an agronomic point of view, introduced new quinoa cultivars showed higher performance than locally cultivated seeds and, furthermore, the use of irrigation and organic amendment has tremendously improved seed yield by double and three times, respectively, compared to rainfed conditions. Nutritional analysis revealed that protein and phosphorus content remained stable after seed pearling while most of the micronutrients content decreased after seed pearling. However, saponins content was reduced by 68% using mechanical pearling compared to 57% using both traditional abrasion and washing. The economic analysis showed that production cost of quinoa seeds could be further decreased using mechanized intensive tools along with irrigation and organic amendment supply. This study revealed several lessons learned from the field experience and proposed several development actions for each value chain component that can be implemented within a national quinoa program.

## 1. Introduction

Today, more than 120 countries around the world cultivate quinoa (*Chenopodium quinoa* Willd.) or try to adapt it to their environmental conditions. The continued expansion of its cultivation in all continents challenges the prejudices of that quinoa is a species, which can only grow in the high plains of the Andes on the shores of Lake Titicaca. After a first boom in quinoa cultivation in the 1990s mainly linked to the demand of vegetarians for products rich in vegetable proteins from organic farming, a second boom in the 2000s was based on the values of fair trade, and, today, we are facing a third quinoa boom at the global level with the production of quinoa in new countries that were not even importing quinoa [1]. Morocco falls into the last category of the country having initiated its cultivation before importing it for its own consumption. These changes on a global scale are such that great transformations in progress in the way quinoa is produced, the networks related to its distribution and in the ways of considering it and incorporating it into various local diets.

The year 2013 has been declared the International Year of Quinoa (IYQ) by the United Nations. This made it possible to recognize the importance of the biodiversity of quinoa and the high nutritional value of its seeds [2]. Within this dynamic, quinoa has been introduced in Morocco since the 1999/2000 season and was considered as an important alternative to traditional crops such as cereals, which are strongly subjected to climate change effects and soil degradation due to salinization making quinoa a judicious solution and potential crop that may contribute to national food security [3]. In this Moroccan context, quinoa is proving to be an interesting solution to limit the risk of agricultural production failure associated with the yield losses observed on traditional cereals cropping systems, which sometimes contribute to soil degradation because of the monoculture practiced in several regions. The fact that quinoa is considered a rustic crop resistant to various abiotic stress makes it a resilient and climate smart crop that could be used for climate change adaptation [4].

In Morocco, quinoa was subjected to several field trials evaluating the performance of introduced cultivars and the effect of various cropping practices on its productivity. First a collection of quinoa accessions was tested for the adaptation goal in the Khenifra region in the year of 2000 resulting in a selection of 14 accessions, which were believed to be tolerant to drought. Then, experiments on quinoa in Morocco were intensified within the SWUP-MED EU funded project (sustainable water use securing food production in dry areas of the Mediterranean region) where quinoa was introduced and tested in several regions including Rehamna, Rabat and Agadir [5]. Secondly, research activities were focusing on testing the effect of several practices on quinoa such as irrigation with saline water [6,7], deficit irrigation [8,9], organic amendment [10], sowing dates [11], use of wastewater for irrigation [12], etc.

At the nutritional level, for some people, quinoa is a new and nutritious food that has recently been found in supermarkets and restaurants and can replace many common grains. Certainly, in many regions of the world, this vision corresponds to reality but it should be known that quinoa was one of the main food crops of the pre-Columbian civilizations of Latin America and remains an important food for the Quechuas and Aymaras settled in rural areas of the Andes, South America. In the Quechua language, quinoa is called chisiya, which means mother grain [13]. Quinoa provides as much energy as foods used in a similar way, such as beans, corn, rice or wheat. It is also an important source of quality protein, dietary fiber, polyunsaturated fatty acids and minerals [14]. Protein content in of quinoa seeds varies between 12% and 20%; however, it is reported as 16% on average [15].

One of the obstacles for quinoa seed valorization is its content in terms of saponins because of their bitter taste and toxic effects, which necessitates their elimination. Several pearling techniques and methods are used to eliminate saponins from the quinoa seeds; the wet technique remains the most used one especially in Morocco combined with preliminary manual abrasion [16].

In this study we provided an evaluation of the agronomic performances of introduced quinoa cultivars grown under different production scenarios and the effect of seed pearling on nutritional and saponin contents. The study also presents a technical and economic analysis of the quinoa production and transformation. Through the conducted investigations the strengths, weaknesses, opportunities, and threats related to the existing quinoa value chain in Morocco were revealed and the lessons learned. Finally, we proposed development perspectives for each value chain component. 

## 2. Results

### 2.1. New High Yielding Chenopodium Quinoa Cultivars Introduction

Table 1 presents obtained results in terms of plant productivity and growth. The data clearly indicate that introduced ICBA (International Center for Biosaline Agriculture) cultivars performed better than other tested varieties in terms of yield while locally produced bulk seeds showed the lowest performances. Irrigation supply and organic amendment have greatly increased the seed yield for all tested cultivars. Under farm conditions, yield was doubled under irrigation supply and tripled under combined irrigation and amendment application. More or less the same effect has been noticed for plant height. Quinoa dry matter responded very well to the amendment application in the case of ICBA-Q3 cultivar while no significant changes were noticed for local bulk seeds. Results obtained for 1000 seed weight indicate that Titicaca cultivar had the highest seed weight and size while Puno cultivar had the lowest. Organic amendment had a notable effect on 1000 seed weight in the case of local bulk seed while no significant difference was obtained for ICBA-Q3 cultivar.

### 2.2. Quinoa Seed Processing

#### 2.2.1. Harvest and Postharvest Machines 

Several mechanized tools were locally developed or adapted to be used for quinoa harvest and post-harvest operations by a private entrepreneur (BenRim farm) as presented in Table 2. Supporting local private entrepreneurs to manufacture those tools was one of the key outcomes of this project.

#### 2.2.2. Quinoa Transformation Pathway

**Figure 1:**
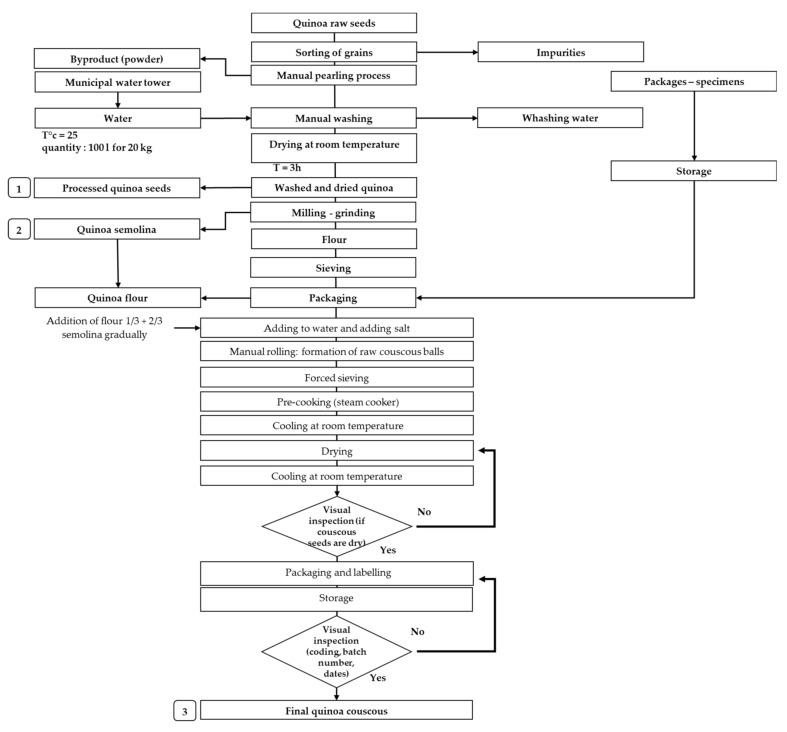
Processing diagram for quinoa based products adapted from the “3rd Millennium” cooperative.

Couscous is a famous Mediterranean dish and widely consumed in Morocco. It is now produced out of quinoa by several women cooperatives in the Rehamna region using both quinoa flour and semolina. The pathway for quinoa-based products processing is described in Figure 1. Quinoa based products described in this study were produced traditionally by women following several steps such as seed pearling, washing and drying to produce processed seeds and milling, grinding and sieving to have quinoa flour and semolina. The key steps of couscous production are manual rolling, forced sieving, precooking and drying. However, 27 steps from raw material reception to final product shipping are required to produce traditional couscous.

#### 2.2.3. Shelling Impacts on the Nutritional and Antinutritional Aspect of Quinoa Seeds

Table 3 shows the nutrients content of raw, processed seeds and quinoa bran for Puno, Titicaca and ICBA-Q5 cultivars. Data clearly indicate that nutrient content varies from cultivar to another. Titicaca processed seeds presents the highest content in terms of proteins, Mg, P, Ca, Zn and Cu, for Puno cultivar the highest content was observed for C and Fe, while ICBA-Q5 processed seeds presents the highest content in terms of K, Na, Mn and Ash.

**Figure 2:**
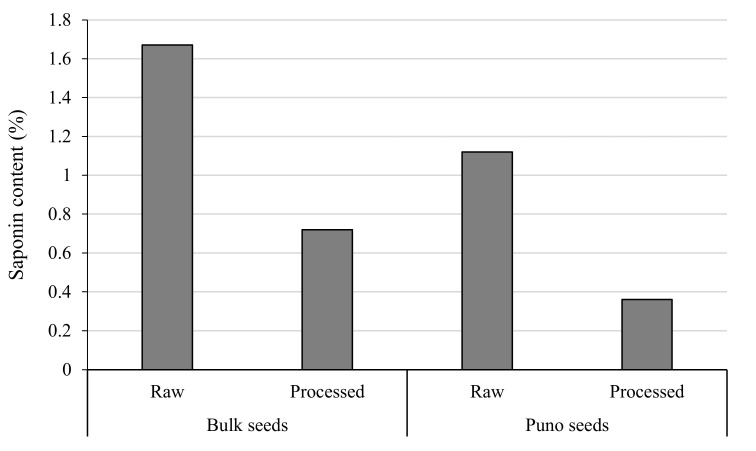
Saponin content (%) of raw and processed bulk and Puno seeds. Bulk seeds were pearled manually and Puno seeds mechanically using quinoa sheller.

Figure 2 presents the saponin content in two different seeds polished using two different methods, bulk seeds polished manually by the women cooperative and Puno seeds polished mechanically using a locally manufactured pearling machine. Obtained results indicate clearly that bulk seeds accumulate more saponins compared to Puno seeds. Pearling using the mechanized tool was shown to be more efficient than manual abrasion as the saponin content was reduced by 68% in the case of Puno and 57% in the case of bulk seeds.

### 2.3. Quinoa Import

**Figure 3:**
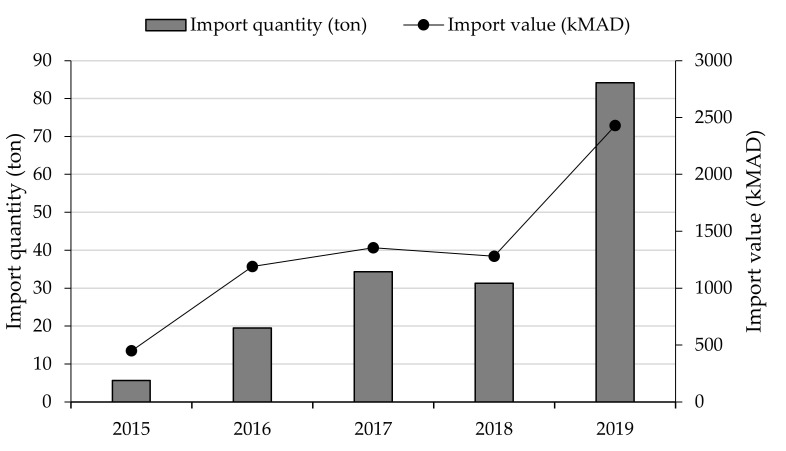
Evolution of quinoa import in Morocco in terms of quantity and value. 1 USD = 9 MAD.

Import of quinoa in Morocco has known a great evolution since 2015 (first year of record) to reach 84 tons in 2019 with a total value of 2.4 million MAD (Figure 3). The average price per kilogram was greatly decreased. In 2015 the average import price was equal to 80 MAD.kg^-1^ while in 2019 it was equal to 29 MAD·kg^−1^ following the worldwide trend in quinoa price as per the Statista database [17].

### 2.4. Economic Analysis of Quinoa Seed Production

#### 2.4.1. Quinoa vs. Traditional Cereals: Production Cost and Net Margin

Data presented in Table 4 clearly indicate that for quinoa cultivation, production cost per kilogram is higher under the rainfed and manual production mode compared to the scenario where irrigation is supplied along with fertilizers and organic amendments. Thus, production cost per kilogram decreased from 27 to 11 MAD and this can be explained by the improved yield and reduced cost attributed mainly to irrigation and fertilization and the use of the mechanized tool for quinoa cultivation. Consequently, the net margin was improved significantly due to input supply and the mechanized tools adoption. The presented data also indicate that quinoa is more remunerating than cultivated cereals as it generates five times and twice the net margin generated by barley and wheat, respectively, grown under the rainfed and mechanized scenario.

#### 2.4.2. Quinoa Production Cost Breakdown

Cost breakdown for quinoa cultivation operations is presented in Table 5. Harvest and post-harvest operations account for the largest part in the overall production cost in the case of rainfed and manual cultivation contributing with 63% in the total production cost. While this percentage was decreased to 50% when mechanized tools for harvest and post-harvest operations were adopted. In the case of the optimized production mode using irrigation and fertilization the part of harvest and post-harvest operations in the total cost was greatly decreased due to high cost attributed to irrigation system depreciation, pumping and fertilizers and was equal to 48 and 26% under manual and mechanized production mode, respectively.

#### 2.4.3. Sensitivity Analysis of Net Margin vs. Production Cost, Grain Yield and Sale Price

**Figure 4:**
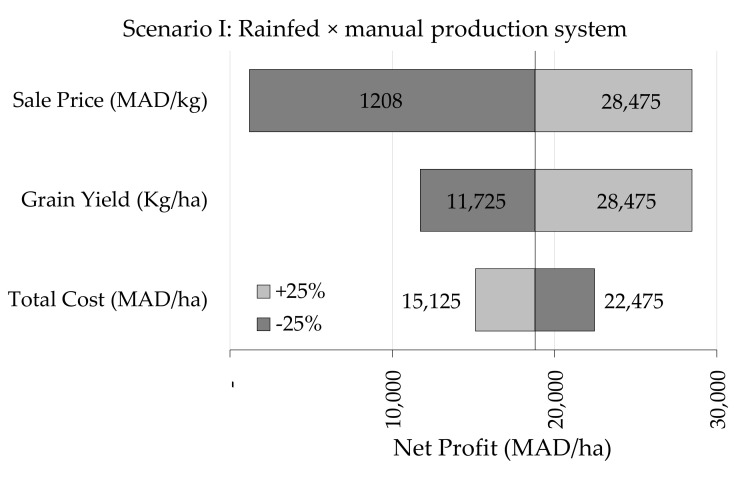

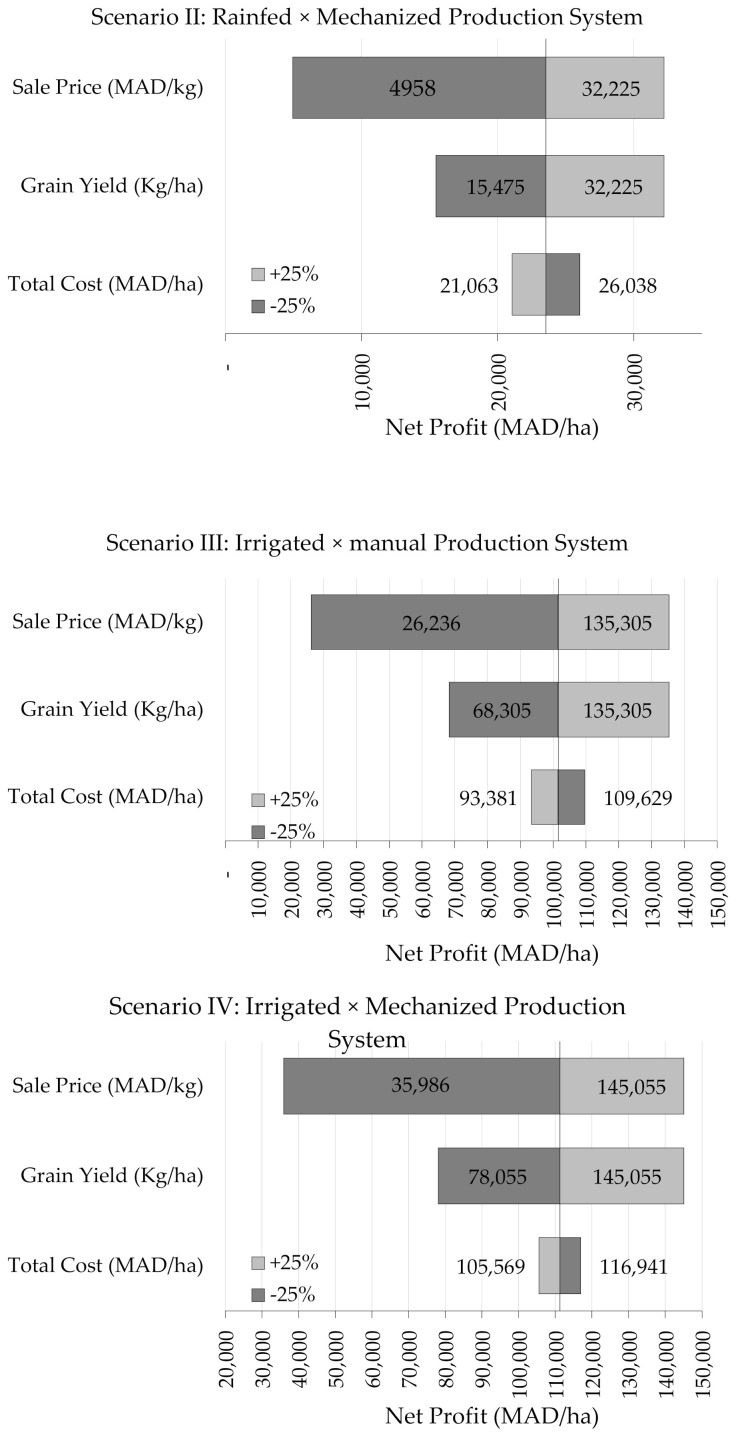
Changes in net profit as responses to ±25% variation in sale price, grain yield and total cost under tested production scenarios. 1 USD = 9 MAD.

Figure 4 presents the sensitivity analysis elucidating the impacts of +25 and −25% changes of total cost, grain yield and sale price on net profit. Results indicate clearly that net profit is affected greatly by sale price as a reduction or increase of 25% in sale price is likely to have the largest impact on net profit either negatively or positively but with a more pronounced effect on the net profit in the case of price reduction. Holding all other variables at their base-value, a 25% reduction in the output price will reduce net profit by 94.2, 82.8, 74.2 and 67.7 percent under scenario I, II, III and IV, respectively. 

#### 2.4.4. Monte Carlo Simulation Analysis (10,000 Iterations)

Monte Carlo simulation performs risk analysis by building models of possible results by substituting a range of values—a probability distribution—for any factor that has inherent uncertainty. It then calculates results over and over, each time using a different set of random values from the probability functions. Depending upon the number of uncertainties and the ranges specified for them, a Monte Carlo simulation could involve thousands or tens of thousands of recalculations before it is complete. In our case the number of iterations made is 10,000 simulating the net profit as affected by changes in market price and yield.

**Figure 5:**
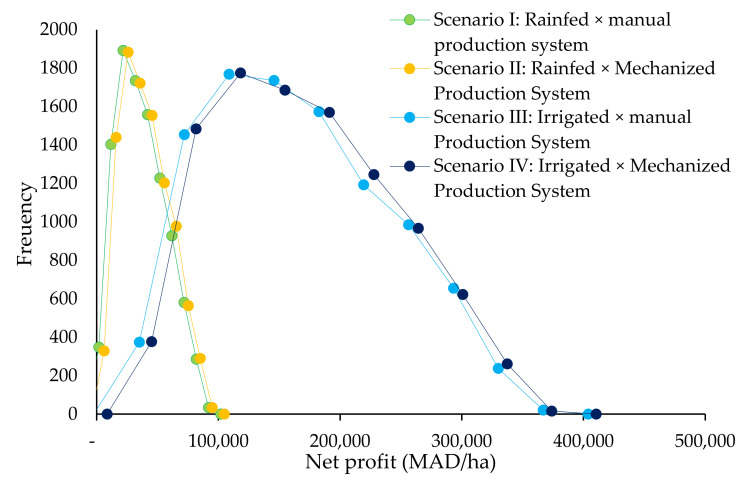
Monte Carlo simulation frequency of net profit as affected by a change in market price and yield. 1 USD = 9 MAD.

The Monte Carlo simulations presented in Figure 5 as frequencies derived after 10,000 iteration of simulation show that the risk of having a financial loss when producing quinoa is about 2.55%, 0.55%, 0.05% and 0% respectively for scenario I, II, III and IV assuming that changes in yield may occur from 200 to 800 kg·ha^−1^ for scenario I and II and from 1000 to 3000 kg·ha^−1^ for scenario III and IV, and allowing for simultaneous variation in price from a lower bond of 30 MAD·kg^−1^ to an upper bond of 120 MAD·kg^−1^.

We also carried out a break-even analysis for each of the four cases to identify values of the key parameters that make revenues equal to the cost of production, and as a result the net gains were zero (Table 6). The estimated break-even points were lower than the lower bonds assumed in the simulations (200 kg·ha^−1^ of yield for scenario I and II, 2000 kg·ha^−1^ yield under scenario III and IV and 30 MAD for the price) indicating higher confidence for the main results presented in Table 5 to remain unaffected. 

#### 2.4.5. Cost Breakdown of Quinoa Based Products 

Table 7 presents cost breakdown for quinoa products such as quinoa couscous, processed seeds and quinoa flour in Morocco. Raw material has the largest contribution to the total production cost for all products with almost 80%, 87% and 85% for quinoa couscous, flour and processed seeds, respectively. Labor cost consisting of women working on different steps of production is also important as it represents more than 13% in the case of couscous while it represents only 1.88 and 3.64% of the total production cost for quinoa flour and processed seeds, respectively. Consequently, among all quinoa products, quinoa couscous remains very expensive compared to other cereals couscous (max market price is equal to 20 MAD·kg^−1^). Obtained data indicate that the women cooperatives producing quinoa products should reduce raw material cost through their own production of quinoa seeds, better planning for quinoa stocks and elaborating the sale contract with quinoa producers for a low price.

### 2.5. SWOT Analysis of the Quinoa Value Chain in Morocco

Table 8 presents analysis of the quinoa value chain in Morocco including its strength. weaknesses. opportunities and threats at different levels.

### 2.6. Lessons Learned

Several lessons were revealed by the present study as presented in Table 9.

### 2.7. Perspectives of Development

With several challenges (drought and salinity) facing staple crops such as cereals in many marginal areas in Morocco. quinoa could be a judicious solution to improve food security and increase farmer’s income. A national program of quinoa in Morocco is becoming necessary and needs to tackle all value chain components. We suggest several actions to improve the quinoa value chain in Morocco summarized in Table 10.

## 3. Discussion

Since its introduction to Morocco in the 2000s. quinoa was seen as a rustic and stress tolerant crop with several potentialities to replace cereals and other traditional crops in the marginal environment of Morocco. Therefore. it was subjected to various trials at the field and pot level in several regions to evaluate its productivity and responses to various stresses. For instance. the finding of this study indicates that quinoa yield was tripled for most of the tested cultivars under full irrigation conditions compared to rainfed. which support the results obtained by Fghire et al. [8] who found that the yield of Puno cultivar (one of the tested cultivars in the present study) conducted in the same study area (Tnin bouchane) was increased by 236% under full irrigation (100% ETp) compared to rainfed irrigation. Our results are in agreement with Geerts et al. [20] who reported that full irrigation increased quinoa yield with 27% compared to rainfed conditions. Contrarily to our case. this low increase percentage is mainly explained by the high amount of rain received (330 mm) for rainfed treatment compared to irrigated treatment (245 mm irrigation + 330 mm rainfall). While in the present study quinoa under farm conditions received only 43 mm of rain during its growing season while the irrigated treatment received 200 mm of irrigation. which obviously explain the tremendous increase of yield and other growth parameters as a response to irrigation.

It is well known that organic matter amendment has a positive effect on crop growth and productivity. In the case of quinoa. very few studies are available evaluating the effect of organic amendment on yield; nevertheless. other crops were cultivated under the organic amendment and showed a positive response [21,22,23]. Our results suggest that the ICBA-Q3 cultivar grown under controlled experimental conditions significantly (*p* < 0.01) responded to the organic amendment only after applying 40 T/ha of manure while no significant difference was observed under a lower dose. While under farm conditions. quinoa yield and plant height were significantly improved under organic amendment application. Our results are in agreement with Hirich et al. [10] who found that that organic amendment of 10 t ha^−1^ and 5 t ha^−1^ significantly increased seed yield by 13% and 3%. respectively. under full irrigation. The yield improvement under organic amendment is explained by a soil content increase in terms of nutrients after mineralization of the organic matter; therefore. the nutrients uptake will be increased. which will result in high plant growth and productivity [24].

Our finding in terms of seed nutrient content indicates that most of the micronutrients content was reduced in the processed seeds due to seed pearling. which means that they are mostly concentrated in the pericarp (bran) as suggested by Konishi et al. [25] and D’Amico et al. [26] who reported that minerals are accumulated in the pericarp (seed out layer) and proteins are mostly accumulated in the endosperm tissues. In terms of mineral content. Konishi et al. [25] reported that phosphorus is mainly localized in the embryonic tissues. which explain why P content in the present study for all tested varieties was higher in the processed seeds and lower in bran. However. in terms of magnesium and potassium our finding indicates high content in the seed bran compared to processed seeds. which disagrees with Konishi et al. [25] who reported that both magnesium and potassium are located in the embryonic tissues. 

One of the limiting factors for quinoa valorization and transformation is its content in terms of saponins. which are mainly concentrated in the pericarp or bran and need to be removed before use [27]. The cultivars we tested in this study are classified as bitter [28]. and the saponins removal level by either mechanical abrasion or manual polishing was not enough to classify the quinoa as sweet. since the saponin content threshold for human consumption is equal to 0.12% according to CODEX [18]. Our results indicate that Puno seeds pearling using mechanical abrasion resulted in 68% reduction in terms of saponin content. which confirm the finding of Hirano and Konishi [29] who reported that quinoa seed pericarps contain 67.6% of the total saponin content in the whole grain while the rest is remaining in the seed endosperm and other internal layer. Our results are in agreement with Mhada et al. [30] who reported that mechanical abrasion allowed the reduction of saponins level from 1.4 to 0.51% for the Puno cultivar. a reduction of 64% of the initial saponin level. Our findings are also in line with Gómez-Caravaca et al. [31] who reported that an abrasion degree of 20% allowed reducing the saponin levels in pearled quinoa (129.8 mg/100 g d.w.) more than 50% comparing with the initial saponin content in whole quinoa (244.3 mg/100 g d.w.).

In Morocco the quinoa market is very limited and still a niche market. In order for the market to expand. huge effort is required to promote for quinoa and a rise in awareness among consumers about its health benefits. Like other countries. such as Turkey. quinoa consumption is limited to those with knowledge of health foods for specific health benefits. including its gluten free status. Quinoa is not a product “consumed by the masses”. but rather one “discovered” by educated. health-conscious consumers [32]. The economic analysis showed that under rainfed conditions production cost at harvest per hectare varies from 6650 to 7900 MAD (739–878 USD) for the mechanized and manual production mode. respectively. While under irrigated conditions the production cost increased to 18.445 and 19.695 MAD·ha^−1^ (2049 and 2188 USD·ha^−1^) for the mechanized and manual production mode. respectively. due to depreciation of the irrigation system. energy and fertilizers input costs. The same trend was reported by Yazar et al. [32] in Turkey who found that the production cost of quinoa was equal to 728 and 1650 USD·ha^−1^ under rainfed and irrigated conditions. respectively. Our results are also in agreement with Mercado and Ubillus [33] who reported that the production cost of quinoa in Peru varies from 676 to 2604 USD·ha^−1^ for traditional rainfed and conventional production system. respectively. with a profitability that varies from 100 to 200% and market price varies from 1.7 to 2 USD·kg^−1^. However. in Morocco the profitability could vary from 150 to 500% due to high quinoa price and the use of intensive production systems (irrigation and mechanized tools).

This study presents a SWOT analysis of the quinoa value chain in Morocco. which revealed that one of the main weaknesses limiting quinoa market expansion in Morocco is the traditional production and valorization and the lack of using intensive production tools. Thus. quinoa price in Morocco remains relatively high above middle class consumer’s purchasing power even at farmgate and only rich people can afford it. This way the local quinoa products with a high price and relatively lower quality could never compete with an imported one. which have usually good quality. The trendy nature of the market for quinoa in Morocco has had both positive and negative aspects. Certainly. growers have benefited from the rising prices that the crop commands. though the various intermediaries may reap more of the profits than the small growers [34]. Another bottleneck in the quinoa value chain is a lack of promotion around quinoa benefits using public channels and social media. which is considered a key point for any new product development [19].

## 4. Materials and Methods

### 4.1. Study Area

**Figure 6:**
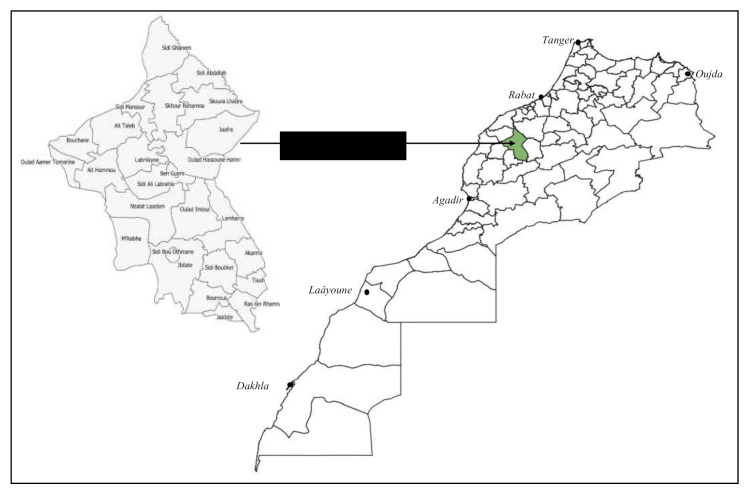
Rehamna province localization (study area).

**Figure 7:**
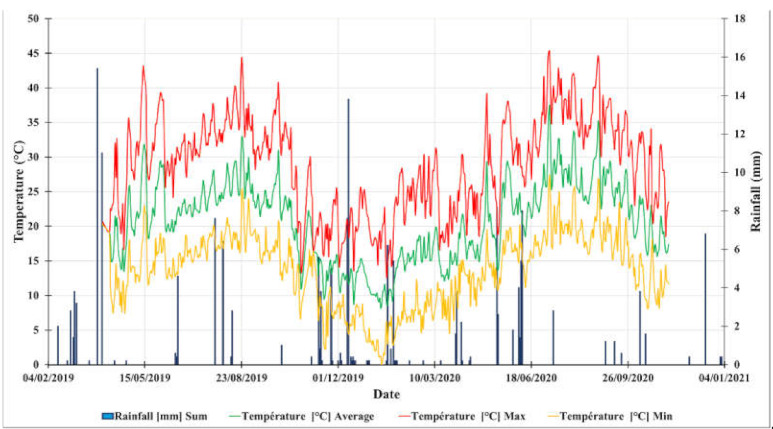
Rehamna climatic conditions taken from the UM6P Experimental Farm meteorological station.

The province of Rehamna is geographically located between Marrakech (South). Settat and El Jadida (North). El Kelaa des Sraghna (East) and Sidi Bennour. Youssoufia to the west (Figure 6). This region is characterized by an average rainfall of 177 mm with an intra and interannual variation (Figure 7). Temperatures are relatively homogenous throughout the zone. with temperatures ranging from 4 to 46 °C. Prevailing winds are from the North-East in winter and from the West in summer. Warm winds (Chergui) are frequent and blow from the East and South. The total agricultural area of the region of Rehamna is 591.125 Ha with an arable land area of 342.500 Ha (35.425 Ha of irrigated area and 307.075 Ha of rainfed area) (DPA Rehamna 2018).

### 4.2. Soil and Water Analysis

Table 11 presents the physicochemical analysis of soils at the UM6P experimental station and farm level. Soil texture is clay loam at the UM6P experimental station and sandy loam at the farm level. Both soils are considered poor in terms of organic matter and rich in terms of potassium. 

According to Table 12. both irrigation waters were slightly saline with more salinity and mineral content obtained for irrigation water at the UM6P experimental station.

### 4.3. Tested Cultivars

In this study ICBA (International Center for Biosaline Agriculture) quinoa cultivars were introduced due to their high adaptation to MENA and Morocco conditions and resistance to drought and salinity [4]. The origins of those cultivars are: low land. Bolivia for ICBA-Q1. Q2 and Q3 and coast. Chile for ICBA-Q4 and Q5. Those cultivars were already introduced and tested in the south of Morocco (Laayoune area) within a previous R&D project and showed high performance under salinity conditions with an average seed yield exceeding 2 t/ha. In addition to ICBA cultivars. two Danish public varieties were tested. Titicaca and Puno. two short cycle varieties widely cultivated in Morocco and showed a good adaptation with higher yield compared to other quinoa accessions [4,5,30,35]. 

### 4.4. Trial Installation

#### 4.4.1. At the Farm Level

An on-farm trial was conducted at the farm level (Tnin Bouchane. 32°14.6267′ N. 8°19.8181′ W. 280 m + MSL (mean sea level)) testing ICBA cultivars (ICBA Q1-Q5) compared to locally cultivated bulk seeds (mixture of L119 and L143 accession) under rainfed. irrigation and irrigation with cow manure amendment (40 T/ha) conditions in a split plot design with 4 replications (plot size was equal to 10 m^2^). Organic amendment was applied along with soil preparation before sowing. Irrigation practices were performed according to farmer usual practices with an irrigation supply of about 200 mm (2000 m^3^/ha) for the whole cropping period. using drip irrigation following evapotranspiration demand according to Allen et al. [36]. Quinoa seeds were sown using a plant density of 8 plants/m² (50 cm between lines and 25 cm between plants). The trials were carried out between 18 February and 30 June 2018.

#### 4.4.2. At the UM6P Experimental Farm

Another trial was carried out in the UM6P experimental farm (Ben Guerir. 32°13.08” N. 7°53.23′ W. 468 m + MSL (mean sea level)) to investigate the performance of six quinoa cultivars including ICBA-Q1. ICBA-Q2. ICBA-Q5. Titicaca. Puno and locally cultivated bulk seeds under Rehamna conditions. The objective of this trial was to assess the productivity of tested cultivars and their adaptation to Rehamna agroclimatic conditions. The trial was conducted in a completely randomized block design with four replications. Plot size was equal to 100 m². Irrigation was applied following the evapotranspiration method according to Allen et al. [36] using parameters from the existing weather station. Irrigation volume supplied was equal to 300 mm (3000 m^3^/ha). Trials were carried out between 21 February and 25 June 2018.

### 4.5. Agronomic Practices and Seed Yield Determination

All trials were subjected to commercial agronomic practices such as soil preparation. preirrigation. weeding (3 times during the growing period). phytosanitary treatments (application of insecticide treatment against caterpillar in the seedling stage) and plant thinning (keeping only one or two plants per sowing hole). Quinoa seeds were sown using a plant density of 8 plants/m² (50 cm between lines and 25 cm between plants).

Seed yield for all trials was determined after maturity. Quinoa panicles were harvested first and dried in open air. Seeds were extracted using manual threshing followed by seed polishing and cleaning.

### 4.6. Seed Pearling

The saponin elimination process remains as a critical operation in seed processing. Recently. many appropriate technologies have been developed to remove saponins to an acceptable threshold without affecting the nutritional properties of the seed.

Puno seeds processed mechanically by Benrim farm were polished using a pearling machine that was locally manufactured for a duration of two minutes. The machine operates on a semi-industrial scale with a transformation capacity of 120 kg·hr^−1^. it is equipped with two motors. the first one is designed to turn a drum with a rotation speed of 750 rpm. The second one is more powerful (3000 rpm) and designed to extract the fine dust produced during the pearling process. The rotating drum is made of 80 cm long perforated stainless steel and has 6 baffles distributed throughout the drum. During seed processing. the speed of rotation and friction (seed-seed. seed-drum and seed-baffles) gradually increased the temperature of the seeds to 35 °C and decreased the moisture from 13 to 10%.

At the level of the women’s cooperatives in Morocco. saponins elimination from quinoa seeds was performed manually with traditional equipment. The majority of valorization units (women’s cooperatives. startups. etc.) used combined operations starting with a dry method (manual abrasion) and finishing by a wet one (washing using water). First. a manual abrasion using a glove against a rough surface (e.g., rubber. sieve) is carried out to eliminate the external coat of the episperm (bran). This manual abrasion operation is time consuming and requires effort; it takes one hour to dehull 6 kg of quinoa seeds. Women’s cooperatives are using a partial manual abrasion in order to avoid losing the embryo and preserve the seed morphological aspect. However, the residual saponin still remains above the CODEX [36] threshold (0.12% of dry matter) and the bitterness perception is still present. Hence. they add a washing step as a supplementary operation to totally eliminate the bitterness. For the washing operation. seeds soaked with water allowing saponins to dissolve. They use a water quantity of 20 L per 5 kg of quinoa seeds for 10–15 min. This quantity is used three times soaking a total of 15 kg of polished quinoa seeds. Finally, the processed quinoa is dried for 5 h.

### 4.7. Chemical Analysis

#### 4.7.1. Nutrient Analysis

Raw and mechanically processed seeds and resulted bran of the Puno variety were used for nutrient content determination following the steps below:

- After weighing. harvested samples were ground to a fine powder using the FOSS CT 293 Cyclotec grinder (Fisher Scientific, Canada).
- The moisture content was measured by drying 100 g of sample at 105 °C for 48 h
- Crude protein was determined using Kjeldahl (Buchi, Switzerland, AACC 46–10) method with a conversion factor of 6.25. Micronutrients were determined after sample mineralization.
- Representative samples (0.25 g) were digested with 7.5 mL of HNO_3_ acid in the DigiPrep System (SCP SCIENCE, France) during two hours at 100 °C.
- After digestion. the solutions were filtered through 45 µm filters. and the filtrates were diluted to 50 mL with deionized water and acidified (2% HNO_3_) in order to undergo the analysis by ICP-OES using Agilent technologies 5110 ICP-OES (Agilent, United States of America) for the elements P. K. Mg. Ca. Cu. Mn. Fe. Zn and B.

#### 4.7.2. Saponin Analysis

In addition to Puno seeds (raw and mechanically processed seeds) used for nutrient content determination. the extraction and quantification of saponin content was performed on locally produced and manually processed by the Bouchane cooperative.

Saponin extraction was performed grinding one gram of dried sample to a fine powder and dissolving in 20 mL of 20% isopropanol. The blend was heated to 86 °C for 20 min for saponin extraction by a microwave-assisted method and filtered (Whatman filter paper) for further quantification. Saponin concentrations were measured by spectrophotometric methods as described by Gianna et al. [37] with minor modifications. The Liebermann-Burchards (LB) reagent was used to quantify saponins. as it is capable of producing a light brown coloration if these compounds are present in a sample. The LB reagent was a 1:5 mixture of acetic acid and sulphuric acid. respectively. Following mixing 1 mL sample solution with 3.5 mL LB reagent. the absorbance at wavelength 580 nm was measured in all samples after 10 min. A calibration curve based on pure quinoa saponins was used for determining the final saponin concentration (mg/mL) in each solution on the basis of absorbance measurements (absorbance = 4.5725 × saponin concentration + 0.0164). The percentage of saponin content was determined on the basis of fresh weight [38]. For nutrient content and saponin determination. three replications have been analyzed. Pictures of grains have been taken using optical microscopy Nikon Eclipse Lv100nd-motorized microscope (Nikon, France) with episcopic/diascopic illumination that enables control of objectives and light intensity from the camera control unit and automatically detects the observation method.

### 4.8. Farmer’s Survey

Quinoa field production cost was determined using face-to-face interviews with farmers cultivating quinoa bulk seeds under different cropping systems (rainfed. irrigated. organic amendment and mechanized). Three farmers from each cropping system were selected and interviewed. The following questions related to production cost breakdown were included in the survey:

- Field operation costs;
- Plowing: deep. superficial;
- Organic amendment: quantity. application;
- Irrigation system: purchase. installation;
- Seeds: quantity. price;
- Sowing: manual. seeder;
- Irrigation: workforce. energy;
- Fertilization: manual. fertigation;
- Weeding: manual. chemical;
- Phytosanitary treatment: insecticide. fungicide;
- Harvest: manual. mechanical;
- Other operations;
- Post-harvest operation costs;
- Yield;
- Panicle drying;
- Threshing: Mechanical. Manual;
- Cleaning: Mechanical. Manual;
- Washing: Mechanical. Manual;
- Seed drying;
- Weighing and packaging;
- Labeling;
- Other operations.

### 4.9. Sensitivity Analysis and Monte Carlo Simulations

Quinoa is not yet a well-established crop in the local production systems. A lack of farmers’ experience and the possibility of a shortage of inputs. especially planting materials. could possibly result in greater yield variability among farmers. The economic performance of quinoa will depend critically on the actual yield performance of different varieties and households’ characteristics and their management practices. which greatly varies among farmers. Beyond production. the markets for quinoa are not well developed. Imperfect and non-competitive markets may fail to clear at competitively determined prices. Poorly functioning markets may therefore pose price risks to the local producers. Hence. such variabilities in yield and potential price volatility may alter the results presented under the base-case scenarios (Table 4). We run simulations of the base-case results to factor in for potential production and price uncertainty.

We first conduct deterministic sensitivity analysis by changing a single parameter. whilst holding all other parameters of the model at their baseline values. In our case. the deterministic analysis was carried out by allowing for a 25% change in yields. prices and total costs to model and assess the sensitivity of net gains for each scenario. While the conventional one-way sensitivity helps determine the scale of impact of a single parameter and its limitation is that it does not proved insights into the probability of such a change (e.g., it does not explain how likely it is for the parameter of interest to take a specific value). Moreover. the deterministic approach fails to take into account the correlation between the values taken by the parameter of interest and other parameters in the model that are held constant [39].

Unlike the deterministic case. simulations allow for stochastic and simultaneous variations and shocks in multiple parameters using the principals of inferential statistics. To evaluate the contemporaneous impact of variations in yields and prices. we then construct a dynamic variant of the model to estimate all possible outcomes given a probabilistic distribution in yields and prices. Using a Monte-Carlo simulation method. we assign multiple values to yields and prices by generating random numbers that follow a symmetric triangular distribution and uniform distribution with lower and upper bonds. respectively. Note that the symmetric triangular distribution is a probability distribution with a probability density function (PDF) shaped like a triangle allowing for central tendency towards the “most-likely or the base-case value”. It therefore gives due weightage to the mean value in the yield with frequent outcomes clustered around the most-likely value. Uniform or triangular distribution assumed for the price variable. on the other hand, allow for the randomly generated number to take any value between the specified upper and lower bonds based on a constant probability. Hence. any value in the specified interval is just as likely and probable [40].

### 4.10. Valorization Cooperative’s Survey

A technical and financial assessment was carried out conducting a diagnostic of the 3rd Millennium” cooperative (a quinoa valorization unit in Rehamna region) in order to assess the technical pathway of quinoa transformation and determine production costs of processed products. The cooperative used Puno variety. which is the most common used by cooperatives and it is provided by the Benrim farm in the Berrechid area where the quinoa price is the most affordable.

### 4.11. Quinoa Import Data

Quinoa import data in terms of quantity and value have been extracted from the change office database [41]. Data were first downloaded searching for quinoa as a keyword and processed using Excel software.

### 4.12. Statistical Analysis

Differences in response variables to applied treatments were assessed using a general linear model with StatSoft STATISTICA 8.0.550 software (StatSoft Inc. Tulsa, OK, USA). Statistical differences were all significant at α = 0.05 or less. The means comparison was based on a one-way ANOVA analysis. 

## 5. Conclusions and Recommendations

In the light of obtained results quinoa was shown to be a potential and resilient crop that could be an alternative to traditional cereals in the marginal area such as the Rehamna region where traditional cereals are not performing well at both the agronomic and economic level. Furthermore. quinoa offers better remuneration and yield under both rainfed and irrigated cultivation. It is also recommended for farmers to adopt mechanized tools for quinoa cultivation and seed processing to reduce production cost and improve their income. The access to those mechanized tools will be easier if farmers are gathered in cooperatives or associations. Quinoa price structuration remains a bottleneck in its value chain in Morocco as quinoa still have a niche market and demand on quinoa products still does not meet the producer’s expectations. Furthermore. production cost of the quinoa-based product such as couscous remains very high due to a high cost of raw material and involved labor force. Therefore. it is recommended for women’s cooperatives valorizing quinoa to have their own quinoa production.

Several scenarios of cost–benefit analysis were conducted to assess the economic viability of quinoa production in Morocco. The results across multiple scenarios consistently indicated that quinoa is highly profitable. yielding a net margin ranging from 21.100 to 111.555 MAD depending on the scenario (e.g., irrigated vs. rainfed and manual vs. mechanized systems). Further sensitivity analysis and simulations were undertaken to analyze the potential impacts of uncertainty in key variables and assumptions. particularly taking into account variability in yield performance. market prices and production costs. The sensitivity analysis showed that output price has the largest and significant impact on the quinoa profitability. However. as is indicated by the results of the simulation. the likelihood of net profit to be negative is neglected with a probability ranging from 0.5 to 2.55% depending on the scenario.

This study revealed several lessons learned from the field experience and proposed several development actions for each value chain component that can be implemented within a national quinoa program. which may be funded within the new Moroccan agricultural development plan called the “green generation”.

The Rehamna Quinoa upscaling project has identified the suitable varieties. and the best production and management practices to maximize yields. In addition. the nutritional analysis of the genotypes with the highest potential was carried out. Seed multiplication of the most promising genotypes has been developed with a private local company to ensure enough seeds are available in the market for the scaling up production. On a global scale. the impact of the project on small farmers and the gender issues has been positive in general. as it secures a minimum revenue for the farmers even in dry years. Therefore. it will be judicious to pursue implementing cutting edge research to collect. screen and identify the quinoa genotypes that have the best potential for wide scale adoption in different agroecological zones and marginal environments.

We recommend then to set up support for all players in the sector through coordination of the quinoa interprofession in Morocco supported by the structures of the Department of Agriculture. Additionally. we need much better organization of the quinoa sector. in order to have more visibility to the consumers. This starts with focusing on more development of quinoa on public awareness and promotion-marketing. More research is needed in reducing saponin content in the different quinoa product. which represent one of the current weak points in the sector and it is necessary to remove this bottleneck as it does affect. today. the quality of the product and the entire value chain. Morocco’s situation within the Maghreb region places it in an advance position in the development of quinoa value chain. This should trigger a promoting cooperation within the Maghreb countries. It is also possible to create a Mediterranean or African network on quinoa to extend ideas and research results.

## Figures and Tables

**Table 1:** Seed yield, plant height, dry matter and 1000 seed weight of quinoa tested in the Rehamna region under several experimental conditions. Different letters (a, b, ab, c) indicate a significant difference according to the Tukey test (*p* < 0.05).

Trial Conditions	Treatments	Seed Yield (t·ha^−1^) of Tested Varieties							
		ICBA-Q1	ICBA-Q2	ICBA-Q3	ICBA-Q4	ICBA-Q5	Titicaca	Puno	Bulk Seeds
Cultivars performance trial under controlled experimental conditions		1.94 b	3.40 a			3.89 a	1.90 b	1.47 b	1.63 b
Organic amendment trial under controlled experimental conditions	0 T/ha			2.20 a					3.16 a
	5 T/ha compost			2.34 a					2.10 b
	10 T/ha compost			2.43 a					2.05 b
	20 T/ha compost			2.31 a					2.40 b
	10 T/ha manure			2.94 ab					1.87 b
	20 T/ha manure			2.50 a					2.23 b
	40 T/ha manure			4.40 b					2.60 ab
Trials under farm conditions	Rainfed	0.74 a	0.90 a	0.71 a	0.51 a	0.63 a			0.54 a
	Irrigated without manure	2.34 a	2.91 a	1.72 ab	1.55 ab	2.70 a			0.85 b
	Irrigated with manure	3.31 a	3.65 a	1.78 b	1.69 b	3.26 a			1.04 b
Plant height (cm)									
Cultivars performance trial under controlled experimental conditions		125.73 a	136.38 a			123.18 a	92.40 b	79.92 b	96.28 b
Organic amendment trial under controlled experimental conditions	0 T/ha			97.35 a					75.75 a
	5 T/ha compost			96.64 a					74.72 a
	10 T/ha compost			101.93 a					81.97 a
	20 T/ha compost			104.83 a					84.94 a
	10 T/ha manure			103.51 a					76.61 a
	20 T/ha manure			108.43 a					88.72 a
	40 T/ha manure			117.06 a					86.11 a
Trials under farm conditions	Rainfed	50.83 a	52.16 a	52.83 a	46.05 a	46.77 a			42.83 a
	Irrigated without manure	115.66 ab	127.5 a	107.58 ab	137.5 a	87.66 b			70.85 b
	Irrigated with manure	109.25 bc	131.9 a	121.75 b	122.75 b	92.91 c			79.75 c
Dry Matter (g·plant−1)									
Cultivars performance trial under controlled experimental conditions		82.83 b	171.47 a			95.70 b	41.40 b	82.54 b	77.19 b
Organic amendment trial under controlled experimental conditions	0 T/ha			101.32 b					75.62 a
	5 T/ha compost			113.33 ab					58.85 b
	10 T/ha compost			119.65 ab					56.66 b
	20 T/ha compost			100.16					61.24 ab
	10 T/ha manure			133.05 a					55.29 b
	20 T/ha manure			94.61 b					61.48 ab
	40 T/ha manure			137.94 a					43.66 c
1000 Seed Weight (g)									
Cultivars performance trial under controlled experimental conditions		2.66 ab	2.58 ab			2.76 ab	3.55 a	1.73 b	2.47 ab
Organic amendment trial under controlled experimental conditions	0 T/ha			5.2 a					3.9 b
	5 T/ha compost			6.5 a					6.0 a
	10 T/ha compost			5.5 a					4.6 ab
	20 T/ha compost			5.4 a					6.1 a
	10 T/ha manure			5.1 a					5.2 ab
	20 T/ha manure			5.7 a					4.5 ab
	40 T/ha manure			6.7 a					5.6 ab

**Table 2:** Harvest and post-harvest machines locally developed.

Machines	Description	Capacity	
Combined harvester	The cereal’s combined harvester was used to harvest quinoa with few adaptations at sieves levels to match quinoa seed size	1.5 ha·hr^−1^	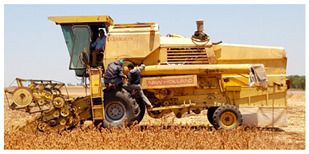
Thresher	The machine can be powered by an electric or a diesel engine. It adopts axial-flow roller	200 kg·hr^−1^	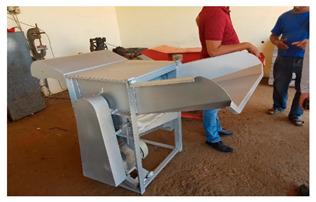
Winnower	Threshing quinoa panicles results in a mixture of grains, small residues and chaffs. The mechanical winnowing consists of using a winnowing fan that creates wind that blows away the lighter chaff, while the heavier grains fall back down for recovery.	150 kg·hr^−1^	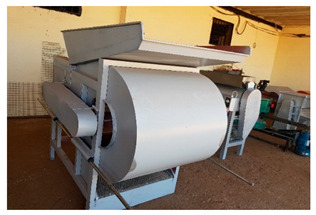
Sheller (pearling machine)	It is equipped with two motors, the first one is designed to turn a drum with a rotation speed of 750 rpm. The second one is more powerful (3000 rpm) and designed to extract the fine dust produced during the pearling process. The rotating drum is made of 80 cm long perforated inox steel and has 6 baffles distributed throughout the drum.	200 kg·hr^−1^	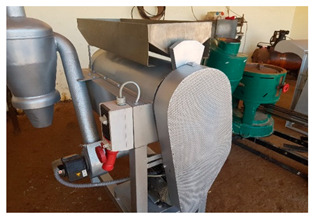

**Table 3:** Nutrients content of raw, processed seeds and quinoa bran for Puno, Titicaca and ICBA-Q5 cultivars.

Cultivar	Products	Protein Content (%)	C (%)	K (%)	Mg (%)	Na (%)	P (%)	Ca (%)	Fe (mg/kg)	Zn (mg/kg)	Cu (mg/kg)	Mn (mg/kg)	Moisture (%)	Ash Content (%)
Puno	Raw seed	14.42	44.70	0.98	0.17	0.09	0.28	0.17	57.35	20.54	5.91	28.22	8	3.74
	Processed seed	15.39	44.35	0.53	0.14	0.06	0.31	0.09	61.55	22.30	4.21	19.73	7	2.30
	Quinoa Bran	14.99	46.48	5.67	0.36	0.11	0.10	0.55	101.64	180.85	13.78	83.78	7	18.00
Titicaca	Raw seed	14.47	43.61	1.33	0.19	0.09	0.35	0.12	67.79	30.30	5.76	32.80	6	3.80
	Processed seed	18.83	43.98	0.72	0.18	0.06	0.43	0.10	51.22	30.94	5.49	23.97	6	2.67
	Quinoa Bran	12.65	42.60	8.87	0.43	0.23	0.19	0.51	521.23	61.98	6.75	109.95	6	17.31
ICBA-Q5	Raw seed	12.18	40.40	1.74	0.20	0.09	0.18	0.10	46.51	26.38	2.48	32.09	7	4.85
	Processed seed	11.07	40.20	1.38	0.16	0.09	0.18	0.05	33.99	27.63	3.80	28.11	7	3.50
	Quinoa Bran	14.61	39.36	6.64	0.77	0.18	0.19	0.54	249.04	68.23	6.02	110.91	7	16.69

**Table 4:** Production cost and net margin of processed quinoa seeds compared to traditional cereals cultivated in the Rehamna region. 1 USD = 9 MAD.

Scenarios	Production System	Yield (kg/ha)	Production Cost (MAD/kg)	Production Cost (MAD/ha)	Net Margin (MAD/ha)
Quinoa Rainfed: Scenario I (manual); Scenario II (mechanized)	Manual	500	26.8	13,400	21,100
	Mechanized	500	19.3	9650	28,850
Quinoa Irrigated with organic amendment and fertilization: Scenario III (manual); Scenario IV (mechanized)	Manual	2000	16.2	32,195	101,805
	Mechanized	2000	11.1	22,445	111,555
Barley under rainfed conditions	Mechanized	2000	1.5	3097	3072
Wheat under rainfed conditions	Mechanized	3000	1.3	3914	6366

**Table 5:** Cost breakdown for quinoa cultivation and seed processing operations. 1 USD= 9 MAD.

Operation	Description	Unit	Production Scenario											
			Rainfed × Manual (Objective Yield: 500 kg/ha)			Rainfed × Mechanized (Objective Yield: 500 kg/ha)			Irrigated with Fertilization × Manual (Objective Yield: 2000 kg/ha)			Irrigated with Fertilization × Mechanized (Objective Yield: 2000 kg/ha)		
			Qty	Unit Cost	Total Cost	Qty	Unit Cost	Total Cost	Qty	Unit Cost	Total Cost	Qty	Unit Cost	Total Cost
Ploughing and soil preparation	Deep	Hour	4	200	800	4	200	800	4	200	800	4	200	800
	Shallow	Hour	2	150	300	2	150	300	2	150	300	2	150	300
Fight against bird’s attack	Manual	Day	15	100	1500	15	100	1500	15	100	1500	15	100	1500
Sowing	Manual/ seeder	Day /Hour	3	100	300	1	250	250	3	100	300	1	250	250
Irrigation and fertigation (energy, depreciation, and fertilizers)		Ha							1	11795	11,795	1	11,795	11,795
Thinning and weeding	First	Day	10	100	1000	10	100	1000	10	100	1000	10	100	1000
	Second	Day	10	100	1000	10	100	1000	10	100	1000	10	100	1000
Harvest	Manual	Day	30	100	3000				30	100	3000			
Threshing	Manual	Day	20	100	2000				20	100	2000			
Seed cleaning	Manual	Day	15	100	1500				45	100	4500			
Seed washing	Manual	Day	10	100	1000				30	100	3000			
Drying and sieving	Manual	Day	10	100	1000				30	100	3000			
Harvest and threshing (combined)	Mechanized	Ha				1	1800	1800				1	1800	1800
Seed pearling	Mechanized	Kg				500	6	3000				2000	2	4000
Total (MAD)					13,400			9650			32,195			22,445

**Table 6:** Break-even analysis. 1 USD = 9 MAD.

Scenario	Yield (kg·ha^−1^)	Price (MAD/kg)	Total Cost(MAD/ha)
Scenario I: Rainfed × manual production system	219.50	29.40	33,500
Scenario II: Rainfed × Mechanized Production System	148.51	19.90	33,500
Scenario III: Irrigated × manual Production System	485.00	16.25	101,505
Scenario IV: Irrigated × Mechanized Production System	339.48	11.37	134,000

**Table 7:** Cost breakdown for quinoa couscous, flour and processed seeds production. 1 USD= 9 MAD.

Quinoa Product	Inputs	Description	Cost (MAD/kg)	%	
Quinoa Couscous	Raw material	Quinoa semolina	21	28.14%	80.40%
		Quinoa flour	39	52.26%	
		Salt	0.004	0.01%	
	Water	Washing	0.02	0.03%	0.04%
		Processing	0.002	0.00%	
	Women’s labor force	Sorting	5	6.70%	13.57%
		Hydration	5	6.70%	
		Packaging	0.12	0.17%	
	Energy	Electricity	0.05	0.08%	0.48%
		Gas	0.3	0.40%	
	Transportation		1	1.34%	1.34%
	Packaging	1 kg package	1.46	1.96%	1.96%
	Depreciation	Dryer	1.64	2.20%	2.22%
		Heat-sealing machine	0.01	0.01%	
	Total		74.62	100%	100%
Quinoa flour	Raw material (Quinoa seeds)		50	87.66%	
	Washing water		0.019	0.03%	
	Energy		0.436	0.77%	
	Labor force		1.073	1.88%	
	Packaging		2.975	5.22%	
	Transport		0.951	1.67%	
	Depreciation		1.580	2.77%	
	Total		57.03	100%	
Quinoa processed seeds 1 kg	Raw material (Quinoa seeds)		50	85.65%	
	Washing water		0.02	0.03%	
	Energy		0.45	0.78%	
	Labor force		2.12	3.64%	
	Packaging		3.12	5.34%	
	Transport		1	1.71%	
	Depreciation		1.65	2.83%	
	Total		58.37	100%	

**Table 8:** SWOT (strength. weaknesses. opportunities and threats) analysis of the quinoa value chain in Morocco.

**Strengths**	**Weaknesses**
*In terms of cultivation*: Quinoa is more profitable compared to cereals. Farmer know-how in terms of cereal production is compatible with quinoa cultivation. Tolerance of quinoa to various stresses that characterize the region including drought and salinity. Quinoa byproducts such as leaves. straw and saponin could be potentially valorized. Low requirement in terms of agricultural inputs (fertilizers. management. pesticides. etc.). *At the gastronomic level:* High nutritional value compared to cereals. Quinoa seeds are gluten free with low content in sugar. which make it optimal food for diabetic and celiac consumers. Fast cooking. Versatility of quinoa-based recipes. Easy to integrate into food habits given its resemblance to locally prepared dishes (soup. boiled rice. couscous. bread. etc.).	*At the production level:* Small production area compared to the potential. Poor organization of producers among those who have adopted quinoa. Quinoa is labor intensive with very few mechanized operations (especially at the post-harvest phase). Problems linked to quinoa establishment at the field level (low germination). Unavailability of good quality seeds. Sensitivity to diseases such as downy mildew. Attacks of birds (during the emergence and maturity stage). Lodging problem in the case of strong winds. High post-harvest costs. *At the valorization and marketing level:* Transformation pathway is not well structured and mastered. Basic marketing channel. Lack of promotion and communication around quinoa-based products. Uncontrolled price formation. Poor product quality. High transformation cost.
**Opportunities**	**Threats**
The Rehamna region presents edaphic-climatic conditions favorable for rustic crops such as quinoa. Policies and development program encouraging the introduction and development of alternative crops (e.g. Green Morocco Plan and Generation Green). Replacement of cactus that was completely devasted by cochineal by quinoa in the Rehamna region. Growing interest by the national and foreign researchers in the adoption of quinoa by farmers. Willingness of national and international development agencies such as the OCP group (Office Chérifien des Phosphates). ONCA (Office National du Conseil Agricole). DPA (Direction Provinciale d’Agriculture). Universities. IDRC (International Development Research Center). FAO (UN Food and Agriculture Organization). etc.. to promote and accelerate the process of adopting quinoa in the area. Availability of national and international agriculture fairs (Salon International d’Agriculture de Meknes) for quinoa products showcase. Increased interest for healthy food consumption by individual consumers and restaurants. Growing quinoa international market. Availability of labours in the rural areas. Remunerative price. Possibility for quinoa product export to the European market.	Competitiveness of local quinoa products compared to imported ones. Substitute products are numerous. High cost and slow process of organic certification. Climatic variability and negative effects of drought and heat waves on quinoa production. Loss of varietal purity and genetic performance due to the use of harvested seeds for many years. Quinoa products supply exceed the demand.

**Table 9:** Lessons learned from the quinoa value chain in Morocco.

Lesson Learned	Action Taken/Needed
**At the Farm Level**	
First. more awareness should be raised among farmers. relevant government entities. private sector and general public about the economic benefits of quinoa and its potential as an alternative crop tolerant to stress and soil–climate conditions of the Rehamna region.	Several training sessions were organized for farmers. women cooperatives and extension agents about quinoa cultivation and its virtues.
In a process of introducing new crop such as quinoa. more technical and economic information is needed to dispel the hesitations of some farmers who are faithful to their usual practices.	Several extension material and brochures about quinoa including a farmer practical guide about quinoa cultivation were produced and shared with farmers and extension agents during organized trainings and workshops.
At the level of production techniques: In spite of the important peasant know-how. quinoa remains a new crop and therefore obeys a logic of adoption. which means categories of progressive farmers (willing to take risk). neutrals (those who see no objection to the introduction of quinoa) and recalcitrant (who are unwilling to question their crop rotation plan). In the first two categories. even a light training in production techniques is necessary.	Field trials conducted by students can serve as a demonstration platform before generalization. The agricultural advisers (institutional partners of the project. ONCA) should act as a link between the results of the experiments and the introduction of quinoa into the farms.
One of the constraints limiting quinoa production is the labor costs. which are estimated by farmers to be excessive compared to the margin generated by the sale of quinoa.	Mechanization of cropping practices is necessary to reduce labor cost including sowing. harvest. threshing. seed pearling and even packaging. Individual small farmers cannot afford those tools. thus. farmer’s organization in cooperatives or associations is considered a judicious option to acquire mechanized tools to be used collectively by the adhered farmers. Several mechanized tools including threshing. winnowing. pearling and seed washing have been locally manufactured and provided to several cooperatives.
Farmers in the Rehamna region usually use the harvested seeds to be sown in the next season for several years. which led to a loss of genetic performance of the initially introduced lines and therefore low germination rate and low performance are usually occurred.	Five introduced varieties (ICBA Q1–Q5) have been registered in the national germplasm catalogue and transferred to a local seed production farm (Benrim Farm) to sustain the production of high quality and homogeneous variety seeds.
In most of farms. quinoa is produced under an organic mode (without application of chemicals) but without certification. Therefore. organic certification is a good option to better valorize quinoa seeds in Morocco and to target international market that require such as certified products (e.g., European market).	The first group of organic quinoa producers has been created in Rehamna in 2018 formed in a first stage by 5 farmers and received organic certification in 2019.
**At the Valorization Level (Women Cooperatives)**	
Seed pearling and saponin removal remains the most critical post-harvest operation as the final quinoa seed quality depends on this step. In most of the cases seed pearling is performed manually. which increase the cost without reaching the saponin content threshold (0.12%) recommended by the CODEX [18].	A pearling machine or sheller (described in the Harvest and Postharvest machines section) has been locally manufactured and preliminary results show good performance of the machine in removing saponin. Several shellers have been distributed to several farmer’s and women’s cooperatives.
The breakdown of quinoa-based product processing costs shows that raw materials account for more than 80% of the total costs. followed by labor.	• The reduction in the cost of raw materials can be achieved by a combination of several practices: ○ The own production of quinoa. which can also improve the tracking of production. ○ The purchase of quinoa grains. in large quantities. during the harvest period. ○ The improvement of quinoa yields per hectare using intensive production system (irrigation. fertilization. etc.).
Weak organization of the women cooperatives linked to several administrative issues mainly due to poor management of the unit. poor distribution of responsibilities. lack of operation’s records. weak coordination. opportunism. decision making. etc.	Training about best practices for cooperative governance and management has been delivered to several women cooperative members to build their managerial and leadership capacity.
**At the Market Level**	
The application of high prices of imported quinoa showcased in supermarkets with small quantities to the locally produced quinoa seeds constitutes a bottleneck in the quinoa marketing in Morocco. Pricing strategy should consider the price of similar products based on other cereal (e.g. couscous). import price. the production cost and the willingness price of the Moroccan consumer. The current price of locally produced quinoa seeds remains high as perceived by the Moroccan consumer.	A marketing study was performed in order to determine the psychological price of quinoa in Morocco [19]. The study indicates that the Moroccan consumer is willing to pay 4–5 USD for one kilogram of processed quinoa seeds.
Promotion and communication around quinoa products and virtues in Morocco still needs to be further developed.	Several promotion and communication activities were organized to showcase quinoa products including: The first quinoa promotion workshop organized in November 2018 inviting a celebrity Moroccan chef to lead a cooking session. Showcase of quinoa products made by women cooperatives in the international fair of agriculture in Meknes in April 2019 (SIAM 2019). Tasting sessions organized in several events including SIAM 2019. Participation in the African Fair of the Social and Solidarity Economy held between 29 October to 5 November 2019 in Senegal by the 3 millennium women cooperative. Inviting national TV and press to elaborate stories about quinoa in Morocco. Project video capsules regularly published in YouTube and other social media channels.
There is a need to develop a specific and unified packaging labeled “Quinoa Rehamna” for all beneficiaries involved in the project including the “gluten free” and “organic” label.	A branding and visual identity document about “Quinoa Rehamna” brand has been elaborated and shared with relevant stakeholders.
For the moment. quinoa producers only commercialize their production at local fairs. weekly rural markets. cereal markets. healthy and organic food shops and individual clients (consumers. restaurants. etc.). Quinoa producers in Morocco could not market their products in the local supermarkets due to a lack of a sanitary certificate for quinoa seed processing and transformation.	Quinoa producers especially women cooperative should first improve their valorization unit to meet the ONSSA (National Food Safety Office) requirements to get the sanitary certificate. which is required by supermarkets. Quinoa producers should also develop partnership with food industries and bakeries to produce quinoa products adapted to the Moroccan context such as couscous. noodles and bakeries and to be marketed at large scale benefiting of their own distribution networks. The organic quinoa producer group recently created should explore export opportunities towards the European market.

**Table 10:** Proposed development actions and involved actors for each value chain components.

Value Chain Component	Development Actions	Involved Actors
Agricultural inputs supply	Registration of quinoa performing varieties Improve the availability of high-quality seeds (seed production systems). Improve the availability of agricultural inputs such as fertilizers. pesticides. etc., especially in remote area (e.g., South of Morocco).	Agricultural input suppliers. Seed production organization and private companies (e.g., SONACOS seed company). Agriculture Ministry departments.
Quinoa production	Adoption of best cropping practices such as: ○ Optimal sowing date. ○ Irrigation supply (deficit or supplemental irrigation). ○ Application of amendments (manure. compost. etc.). ○ Application of fertilizers. ○ Increase sowing density to reduce plant lodging. ○ Plant thinning and hilling. Plant protection: ○ Protection from bird attacks (Maghreb lark) at the early stage (emergence and seedling). ○ Protection against caterpillars at early stage. ○ Treatment against downy mildew. ○ Use of a wind-break to protect quinoa from strong wind and reduce lodging. Harvest: ○ Plant harvest at optimal stage to avoid grain loss. ○ Use of mechanized tool for harvest (e.g., adapted combined harvester). Continuous training for farmers about best cropping practices.	Farmers. Farmer’s cooperatives and associations. Agriculture Ministry departments. Research and development institutions.
Quinoa transformation and valorization	Use of a mechanical sheller for saponin removal. Respect of the transformation line and separation in time and space for gluten free products. Improve women cooperative’s management skills. Improve packaging and storage conditions. Build the technical capacity of women in the best quinoa transformation practices.	Women cooperatives. Agriculture Ministry departments. Research and development institutions. Food industries.
Marketing and distribution	Elaborating marketing study for specific quinoa products. Use of social media and influencer to promote quinoa products. Develop the “quinoa Rehamna” label. Develop a sale contract with super and hypermarkets. Target groceries and weekly markets. Target E-commerce platforms. Develop contracts with food industries. Target direct clients such as restaurants. hotels. individuals. etc. Participation in food and agricultural fairs.	Farmers. Farmer’s cooperatives and associations. Women cooperatives. Agriculture Ministry departments. Research and development institutions. Supermarkets. Retailers.
Consumption	Conduct promotion activities including: ○ Video capsules in TVs. radios and social media. ○ Organize tasting sessions in food fairs and supermarkets. Develop promotion materials such as brochures. posters. etc.	Women cooperatives. Research and development institutions. Supermarkets. Retailers.
Research and development	Further research and development activities are needed in the following subjects: Quinoa breeding for new adapted and stable varieties. Optimization of quinoa fertilization. Mechanized sowing. Harvest and post-harvest mechanized tools development. Development of saponin removal methods and techniques. Valorization of quinoa byproducts (saponins). Market assessment.	Farmers. Farmer’s cooperatives and associations. Women cooperatives. Agriculture Ministry departments. Research and development institutions.

**Table 11:** Physicochemical analysis of soil at the UM6P experimental station and farm level.

Parameter		Unit	At UM6P Experimental Station Level	At Farm Level	Analysis Method
Granulometric composition	Clay	%	32.15	6.03	NF X 31-107
	Silt	%	26.45	23.54	NF X 31-108
	Sand	%	42.34	70.46	NF X 31-109
pH-water			8.38	7.9	NF ISO 10390
Electric Conductivity (EC) 1/5 at 25 °C		mS·cm^−1^	0.19	0.1	NF ISO 11265
Total limestone (CaCO_3_)		%	4.83	0.2	NF EN ISO 10693
Organic matter		%	1.07	0.8	NF ISO 14235
Phosphorus (P_2_O_5_)		Mg·kg^−1^	17.15	43.45	NF ISO 11263
Potassium (K_2_O)		Mg·kg^−1^	455.45	216	NFX 31-108
Ammonium (NH_4_^+^)		Mg·kg^−1^	6.45	10.25	NFX 31-109
Nitrate (NO_3_^−^)		Mg·kg^−1^	7.91	12.50	NFX 31-110

**Table 12:** Physicochemical analysis of irrigation water at the UM6P experimental station and farm level.

Parameter	Unit	At UM6P Experimental Station Level		At Farm Level		Method
pH		9.28	7.91		NM ISO 10523	
Electric conductivity at 25 °C	mS·cm^−1^	2.50	1.44		IM ISO 7888	
Dry extract	g.l^−1^	1.60	0.92		NM 03.7.027	
Cations						
Potassium (K^+^)	meq.l^−1^	0.22	0.01		Continuous Flow Analysis (CFA)	
Sodium (Na^+^)		14.67	5.40		Continuous Flow Analysis (CFA)	
Calcium (Ca^2+^)		3.50	2.31		Continuous Flow Analysis (CFA)	
Magnesium (Mg^2+^)		6.80	6.45		Continuous Flow Analysis (CFA)	
Anions						
Chloride (Cl^−^)	meq.l^−1^	17.44	7.17		Continuous Flow Analysis (CFA)	
Carbonate (CO_3_^2−^)		2.40	0.17		NM ISO 9963-1	
Bicarbonate (HCO_3_^−^)		2.60	0.77		NM ISO 9963-2	

## Data Availability

The data generated within this work are open access and available to be shared with interested persons according to signed agreement with IDRC.
